# **β**-Glucosidases from the Fungus *Trichoderma*: An Efficient Cellulase Machinery in Biotechnological Applications

**DOI:** 10.1155/2013/203735

**Published:** 2013-08-01

**Authors:** Pragya Tiwari, B. N. Misra, Neelam S. Sangwan

**Affiliations:** ^1^Metabolic and Structural Biology Department, CSIR-Central Institute of Medicinal and Aromatic Plants (CSIR-CIMAP), P.O. CIMAP, Lucknow 226015, Uttar Pradesh, India; ^2^Department of Biotechnology, UP Technical University, Lucknow 226021, Uttar Pradesh, India

## Abstract

**β**-glucosidases catalyze the selective cleavage of glucosidic linkages and are an important class of enzymes having significant prospects in industrial biotechnology. These are classified in family 1 and family 3 of glycosyl hydrolase family. **β**-glucosidases, particularly from the fungus *Trichoderma,* are widely recognized and used for the saccharification of cellulosic biomass for biofuel production. With the rising trends in energy crisis and depletion of fossil fuels, alternative strategies for renewable energy sources need to be developed. However, the major limitation accounts for low production of **β**-glucosidases by the hyper secretory strains of *Trichoderma*. In accordance with the increasing significance of **β**-glucosidases in commercial applications, the present review provides a detailed insight of the enzyme family, their classification, structural parameters, properties, and studies at the genomics and proteomics levels. Furthermore, the paper discusses the enhancement strategies employed for their utilization in biofuel generation. Therefore, **β**-glucosidases are prospective toolbox in bioethanol production, and in the near future, it might be successful in meeting the requirements of alternative renewable sources of energy.

## 1. Introduction 

*β*-glucosidases are members of cellulase enzyme complex and are promising candidates in biotechnological applications. Fungal species belonging to genus *Trichoderma* are ubiquitous in nature and classified as imperfect fungi due to absence of sexual reproduction [1]. *Trichoderma* is a saprophyte and produce diverse enzymes, a particular strain being specific for a certain type of enzyme. For example, *T. reesei* is used for cellulase and hemicellulase production, *T. longibratum* is used for xylanase, and *T. harzianum *is used for chitinase [2]. The cellulase system in *T. reesei* constitutes the combined activity of three enzymes: cellobiohydrolase, endo-*β*-glucanase and *β*-glucosidases, respectively. Cellobiohydrolases (EC 3.2.1.91) degrade cellobiose residues from the nonreducing end of the glucan, endo-*β*-glucanase (EC 3.2.1.4) catalyzes the breakdown of internal *β*-1,4-linkages, while *β*-glucosidases (EC 3.2.1.21) hydrolyze cellobiose to two molecules of glucose [3]. The conversion of cellulose to glucose is regarded as the rate limiting step in the production of biofuels from lignocellulosic materials, due to high cost of cellulases and their low efficiencies. 

*β*-glucosidases, also named as (*β*-D-glucoside glucohydrolase, EC 3.2.1.21), catalyze the hydrolysis of the *β*-glucosidic linkages such as alkyl and aryl *β*-glucosides, *β*-linked oligosaccharides as well as several oligosaccharides with release of glucose [4, 5]. *β*-glucosidases are prominent class of enzymes and catalyze cellulose degradation acting synergistically with cellobiohydrolase and endoglucanase, respectively [6]. The specificity of *β*-glucosidases is variable towards different substrates depending on the enzyme source. The enzyme is ubiquitously present in nature and found in bacteria [7], fungi [8], yeasts [9], plants [10–13], and animals [14], respectively. 

 Some *Trichoderma *species amongst cellulolytic fungi have strong cellulose-degrading properties and therefore their cellulase systems have been widely studied. In *T. reesei*, the maximum production of cellulase component is of cellobiohydrolases I (CBHI) which is 60% of the total secreted protein [15], while cellobiohydrolases II (CBHII) and endoglucanases accounts for 20 and 10% of the total secreted protein and this is a major limitation in cellulose saccharification by cellulases [16]. 

 The mechanism of catalysis includes the degradation of cellobiose to glucose resulting in cellulose saccharification and release of the two enzymes from cellobiose inhibition [17, 18]. The enzymes are widely distributed in microbes, plants, and animals and play important roles in biological processes [19]. *β*-glucosidases, particularly from microorganisms, play a significant role in cellulose saccharification. However, microbes which produce the enzyme in low quantities lead to inefficient degradation of cellulose. While in microorganisms, *β*-glucosidases are involved in degradation of cellulose as compared to synthesis of beta-glucan during cell wall development, fruit ripening, defense mechanisms, and pigment metabolism [20, 21]. However, *β*-glucosidase-1 (BGL1) from *T. reesei* hyperproducing strain is produced in very small quantities. Over expression strategies in *T. reesei* or additional incorporation of *β*-glucosidase from other sources could be a possible option for enhancing and optimizing *β*-glucosidases mediated cellulose degradation. The products, cellobiose generated by endo- and exoglucanase act as inhibitors of both enzymes and removed by the action of *β*-glucosidases [22]. 

Several studies on *β*-glucosidases, time and again have highlighted their importance in biotechnological applications. Woodward and Wiseman reviewed the research on the fungal enzymes till 1982 [23]. Further, the enzymes from yeast were studied by Leclerc and coworkers [24] and thermostable *β*-glucosidases from mesophilic and thermophilic fungi [25]. Recently, molecular cloning studies on *β*-glucosidases were performed by Bhatia and colleagues [26]. Several other studies report on the isolation, cloning, and purification of *β*-glucosidases [27, 28].

 With the present trends in rising the importance of *β*-glucosidases in industrial applications, this review is an update on fungal *β*-glucosidases particularly from *Trichoderma* species, an overview of their increasing significance, classification of the enzymes, their structure and properties, and also their prospective role in biotechnological applications. Furthermore, *β*-glucosidases may serve as a promising tool in meeting the energy crisis by generating an alternative renewable source of biofuels production in future.

## 2. Phylogenetics and Characteristics of *Trichoderma* Fungus 

The genus *Trichoderma* is the best studied among fungi due to its biotechnological prospects and applications. The first report pertaining to the fungus *Trichoderma *dates back to 1794 [29]. Bioinformatics approaches such as oligonucleotide barcode (TrichOKEY) and a similarity search tool (TrichoBLAST) are mostly used in *Trichoderma* studies and can be accessed online at www.isth.info [30, 31]. Phenotype microarrays are the more reliable technique for the identification and characterization of newly isolated *Trichoderma *spp.

 The cellulases produced from the *Trichoderma* species are important industrial products for biofuel production from cellulosic waste. *Trichoderma* species is widely present on cellulosic materials and results in their degradation [32]. At present, 165 records for *Trichoderma* are available in the Index Fungorum database (http://www.indexfungorum.org/Names/Names.asp). The international subcommission on *Trichoderma* includes 104 species characterized at the molecular level (http://www.isth.info/biodiversity/index.php). *Trichoderma *is among the most extensively used fungus species in industrial applications. The whole genome sequencing of the three strains, *T. reesei*, the industrial strain [33] (http://genome.jgi-psf.org/Trire2/Trire2.home.html), *T. atroviride* and *T. virens*, two other important biocontrol species (http://genome.jgi-psf.org/Trive1/Trive1.home.html) is under progress. The results showed that although *T. reesei* is considered as an important industrial strain for cellulose degradation, its genome consists of fewer genes encoding hemicellulolytic and cellulolytic enzymes [34]. 

 Several species of *Trichoderma*, namely, *T. reesei*, *T. atroviride*, *T. virens*, *T. asperellum*, *T. harzianum*, *T. citrinoviride*, and *T. koningii* are considered important and used in various industrial applications. Studies on *β*-glucosidases from *Trichoderma* species ranging from protein purification and characterization and overexpression in different fungal strains to site-directed mutagenesis and molecular biology studies have been summarized in Table 1.

## 3. Structure of ***β***-Glucosidases

 With the increasing significance of *β*-glucosidases and their application in industrial biotechnology, efforts have been made to isolate a wide range of *β*-glucosidases from different sources and also, on the improvement of enzyme activity and thermostability. The structure of *T. reeseiβ*-glucosidase 2 (TrBgl2) has been elucidated by Lee and coworkers in 2012 [35] with a PDB code-3AHY. The structure of TrBgl2 consists of Glu165 as the catalytic acid/base and Glu367 as the catalytic nucleophile [36] and utilizes a *β*-retaining mechanism for its activity. The enzyme adopts a (*α*/*β*)_8_-TIM barrel fold typical of GH1 enzymes, with the active site including a deep pocket from enzyme's surface to the barrel core of the protein. Two conserved motifs, namely TFNEP and VTENG comprising of catalytic acid/base E165 and catalytic nucleophile E367 are situated opposite to each other at the bottom of active site. The amino acid residues supposed to be involved in substrate binding are as follows: glycone-binding residues: Q16, H119, W120, N164, N296, W417, N422, E424, W425, T431, and F433; aglycone binding residues: C168, N225, F228, Y298, T299, and W339) [36]. Mutational studies were carried out to determine the functional role of amino acids in active site. Two mutants (F250A and P172L/F250A) with increased enzyme catalytic efficiency and two mutants (L167W and P172L) with enhanced thermostability were generated [35]. Structural studies using bioinformatics approaches, are a key platform to decode the structural aspect of *β*-glucosidases and to understand its catalytic mechanisms.

## 4. Classification and Properties of Fungal ***β***-Glucosidases

*β*-glucosidase are classified in glycosyl hydrolase family, and include 132 families according to CAZY web server [47]. *β*-glucosidases from archeabacteria, plants, and mammals are found in family 1 and usually exhibit *β*-galactosidase activity while family 3 consists of *β*-glucosidases from bacteria, fungi and plants [48]. Family 1 and family 3 include retaining enzymes that hydrolyze the substrates with retention of anomeric carbon via a double-displacement method [49, 50]. 

Cellulose constitute one of the most abundant organic biopolymers on earth, and the cleavage of glycosidic bonds plays a crucial role in a wide range of biological processes in all living organisms. *β*-glucosidases comprise of a major enzyme group and are classified into 1st and 3rd families and hydrolyze either *S*-linked *β*-glycosidic bonds (myrosinase or *β*-D-thioglucoside glucohydrolase, EC 3.2.3.1) or *O*-linked-glycosidic bonds (*β*-D-glucoside glucohydrolase, EC 3.2.1.21) [51]. 

 Based on substrate specificity, *β*-glucosidases are classified in three classes: class I (aryl *β*-glucosidases), class II (true cellobiases), and class III (broad substrate specificity enzymes). Mostly, *β*-glucosidases belong to class III with diverse catalytic mechanisms including cleavage of *β* 1,4; *β* 1,6; *β* 1,2 and *α* 1,3; *α* 1,4; *α* 1,6 glycosidic bonds [26, 52]. The enzymes exhibit functional diversity in terms of substrate specificity and no specific catalytic mechanism has been observed. However, the fungal enzymes are classified on the basis of their relative activities toward cellobiose and P(O)NPG into two groups, namely, (1) cellobiases—enzymes which have higher activity towards cellobiose, and (2) Aryl-*β*-glucosidases—higher relative activities towards P(O)NPG than cellobiose or negligible activity towards cellobiose. These are further classified according to their affinities towards cellobiose and P(O)NPG into three groups: (1) *β*-glucosidases with higher affinities for P(O)NPG, (2) *β*-glucosidases which show higher affinity (lower *K*_*m*_) for cellobiose and (3) *β*-glucosidases with affinities (*K*_*m*_) similar for both substrates [53]. The values of *K*_*m*_ range from 0.031 (*Neocallimastix frontalis*) [54] to 340 mM (*β*-glucosidase II from *P. infestans*) [55] for cellobiose and from 0.055 mM (*Stachybotrys atra*) [56] to 34 mM (*β*-glucosidase II from *P. infestans*) [55] for P(O)NPG substrate. 

*β*-glucosidases are biologically important enzymes and catalyze the transfer of glycosyl group between oxygen nucleophiles. Also, these enzymes exhibit activity for both natural (plant) or synthetic aryl-glucosides and a variety of aglycons [53]. A *β*-glucosidase purified from *A. niger* showed catalytic activities towards the disaccharides gentiobiose (*β* 1–6), sophorose (*β* 1-2), laminaribiose (*β* 1–3), and salicin (salicyl-glucose) [57]. The glucosidase from *P. herquei*, G1 *β*-glucosidase demonstrated relative activities of 82.7 and 70.3% toward gentiobiose and salicin (100% for PNPG) while the G2 isoenzymes are 8.7 and 54.5%, respectively [58]. This indicates that variations exist between enzymes from different species as well as between isoenzymes of the same microorganism. These enzymes possess high activity towards oligosaccharides with *β* (1→4) linkages; several studies indicated a higher activity towards glucans with *β* (1→2) and *β* (1→3) linkages. Examples include enzymes from *T. koningii *[59] and *A. fumigates *[60] with activity towards sophorose and laminaribiose than cellobiose. Although, the enzymes exhibit greater variability towards *β*-1,2/1,3 *β*-glucans, aryl-glucosides, and cellooligosaccharides, these enzymes are specific for *β*-anomeric configuration (exception *β*-glucosidase from *Thermomyces lanuginosus, *shows *α*-glucosidase activity) [5]. 

 Mainly, *β*-glucosidases display optimum pH over the range 4.0 to 5.5 but enzyme activity has also been observed in low pH range (pH 2.5) to very high range (pH 8.0). The optimum temperature range for enzyme activity is from 35° to 80°C. The extracellular *β*-glucosidases from mesophilic fungi are thermostable enzymes (up to 60°C). Example includes a *β*-glucosidase purified from *T. reesei *QM 9414 strain which shows high stability of 50–55°C [61]. Several reports indicated the role of the carbohydrates in thermostability of the enzymes as cellulases are mostly glycoproteins. Examples are *β*-glucosidases I, III, and IV from *T. emersonii* [62] and *β*-glucosidases of *Mucor miehei* [63]. 

 Glucono-*δ*-lactone is a potent competitive inhibitor of many *β*-glucosidases, and values of Ki ranging from 0.0083 *μ*M to 12.5 mM have been reported [53]. Steric similarities between the enzyme-bound substrate and Glucono-*δ*-lactone might explain the competitive inhibition by this compound [64]. Other inhibitors of the enzyme include nojirimycin and deoxy nojirimycin [65] and heavy metals such as Hg^2+^, Cu^2+^, Pb^2+^ and Co^2+^, and *p*-chloromercuribenzoate [66]. 

*β*-glucosidases from *T. reesei* are found bound to the cell wall or cell membrane or in supernatants with pI ranging from 4.4 to 8.7. In *T. reesei*, most of the enzyme is bound to the cell wall [67] during fungal growth and therefore low quantities of *β*-glucosidase are secreted into the medium [68]. Kubicek [69] reported that the membrane-bound *β*-glucosidase plays a role in the formation of sophorose which acts as a potent inducer of cellulases. Studies also indicated that the enzyme may act in cell-wall metabolism during conidiogenesis and therefore, not really a true component of cellulolytic enzyme system [67]. Inglin and coworkers [70] isolated an intracellular *β*-glucosidase and postulated that the enzyme might be involved in transportation across cell membrane as a proenzyme and in metabolic regulation of cellulose induction. 

## 5. Studies on ***β***-Glucosidases from *Trichoderma* Species

Numerous studies on *Trichoderma *have indicated its importance in biotechnological perspectives. Several molecular biology and biochemical techniques have reported the improved isolation of *β*-glucosidases from different species of *Trichoderma* namely *T. reesei* [37–41, 43, 71], *T. atroviride *[72],* T. harzianum* [42, 44], *T. viride* [46, 73], *T. koningii* [59], and *T. citrinoviride* [28], respectively (Table 1). Some of the key studies on *β*-glucosidase from *Trichoderma *fungus are as follows.

### 5.1. Protein Purification

Biochemical studies resulting in purification and characterization of a *β*-glucosidase from Type C-4 strain of *T. harzianum* was performed by Yun et al. [42]. A *β*-glucosidase with high cellulolytic activity was purified to homogeneity through Sephacryl S-300, DEAE-Sephadex A-50, and Mono P column chromatographic steps. SDS-PAGE analysis revealed that the protein was a monomer with a molecular mass of 75 kDa. The enzyme properties were established in terms of optimum activity at pH 5.0 and 45°C. *p*-Nitrophenyl-**β**-D-cellobioside and *p*-Nitrophenyl-**β**-glucopyranoside served as substrates and glucose and gluconolactone acted as competitive inhibitors, respectively. Similar studies by Chandra and coworkers [28] reported the homogenous purification, kinetics, and MALDI-TOF assisted proteomic analysis of an extracellularly secreted *β*-glucosidase of *T. citrinoviride*. The enzyme had a molecular weight of 90 kDa, consisted of a single polypeptide chain, optimal activity at pH 5.5 and 55°C. Further, the enzyme was not inhibited by glucose (5 mM) and possess transglycosylation activity (catalyze conversion of geraniol to its glucoside).

 Another study reported the comparative kinetic analysis of two fungal strains, *β*-glucosidase from *Aspergillus niger* and BGL1 from *T. reesei* through an efficient FPLC technique. 95% purification was obtained for BGL1 from *T. reesei* and cellobiose was used as substrate for kinetic characterization of the enzyme. The study revealed that *β*-glucosidase, SP188 from *Aspergillus niger* (*K*_*m*_ = 0.57 mM; *K*_*p*_ = 2.70 mM), has a lower specific activity than BGL1 (*K*_*m*_ = 0.38 mM; *K*_*p*_ = 3.25 mM) and more sensitive to glucose inhibition. Furthermore, a Michaelis-Menten model was generated and revealed comparative substrate kinetics of *β*-glucosidase activity of both enzymes [3]. Chirico and Brown [45] purified a *β*-glucosidase from the culture filtrate of *T. reesei *QM9414 strain to homogeneity and the purified enzyme exhibited activity towards cellobiose, p-nitrophenyl *β*-D-glucopyranoside and 4-methylumbelliferyl *β*-D-glucopyranoside.

 A new type of aryl-*β*-D-glucosidase with no activity towards cellobiose was isolated and purified from a commercial cellulase preparation derived from *T. viride*. The purification techniques included Bio-Gel gel filteration, anion exchange on DEAE-Bio-Gel A, cation exchange on SE-Sephadex, and affinity chromatography on crystalline cellulose. The enzyme had a molecular weight of 76,000 Dalton and showed high activity with on* p*-nitrophenyl-*β*-D-glucose and *p*-nitrophenyl-*β*-D-xylose and moderate activity towards crystalline cellulose, xylan, and carboxymethyl cellulose [46]. 

### 5.2. Genomics Studies

#### 5.2.1. Promoter Analysis

 Although *T. reesei* have been explored extensively for cellulase production, the major limitations are the low *β*-glucosidase activity and inefficient biomass degradation, respectively. The xyn3 and egl3 promoters were used to enhance the expression of *β*-glucosidase 1 (BGL1) through homologous recombination. The recombinant strains showed 4.0- and 7.5-fold higher *β*-glucosidase activity under the control of egl3 and xyn3 promoters as compared to native strains. Furthermore, Matrix assisted laser desorption ionization-time of flight (MALDI-TOF) mass spectrometry determination revealed that BGL1 was over expressed. The increased level of BGL1 was adequate for cellobiose and cellotriose degradation [74]. 

#### 5.2.2. Mutational Studies

The mutants of *T. reesei* capable of cellulase overproduction have been considered significant and economical for saccharification of pretreated cellulosic biomass [75]. Low BGL activity in *T. reesei *results in cellobiose accumulation leading to reduced biomass conversion efficiency and cellobiose-mediated product inhibition of CBH I (Cel7A) [76]. Exogenous supplementation of BGL in *T. reesei *cellulase preparations has been used as an alternative strategy to overcome this problem [77, 78].

Nakazawa and coworkers [38] constructed a recombinant *T. reesei* strain, X3AB1 that was capable of expressing an *Aspergillus aculeatus* *β*-glucosidase 1 with high specific activity under xyn3 promoter control. The study involved the isolation and harvesting of the culture supernatant from *T. reesei* X3AB1 grown on 1% Avicel (as carbon source). It exhibited 63- and 25-fold higher *β*-glucosidase activity against cellobiose compared to those of the parent strain PC-3-7 and *T. reesei *recombinant strain expressing an endogenous *β*-glucosidase I, respectively. The study further demonstrated that xylanase activity was 30% less when compared to due to the absence of xyn3 promoter. X3AB1 strain when grown on 1% Avicel-0.5% xylan medium, produced 2.3- and 3.3-fold more xylanase and *β*-xylosidase, respectively, than X3AB1 grown on 1% Avicel.

Furthermore, a mutant strain of *T. citrinoviride* was developed by multiple exposures to ethidium bromide and ethyl methyl sulphonate [79]. The mutants secreted FPase, endoglucanase, *β*-glucosidase and cellobiase 0.63, 3.12, 8.22, and 1.94 IU mL^−1^ which was found to be 2.14-, 2.10-, 4.09-, and 1.73-fold higher compared to the parent strain. Further studies indicated that under submerged fermentation conditions, glucose (upto 20 mM) did not led to inhibition of enzyme production. Comparative fingerprinting revealed the presence of two unique amplicons suggesting genetic uniqueness of the mutants. 

#### 5.2.3. Molecular Cloning and Heterologous Expression

 A novel fungal *β*-glucosidase gene (bgl4) and its homologue (bgl2) have been cloned from *T. reesei* [43]. This enzyme reportedly showed homology with plant *β*-glucosidases classified in *β*-glucosidase A (BGA) family. The BGL2 protein from *T. reesei *showed an amino acid composition of 466 on SDS PAGE and exhibited 73.1% identity with *β*-glucosidase from fungus *Humicola grisea.* Both the genes have been expressed in *Aspergillus oryzae *and purified. Furthermore, *β*-glucosidases of *Humicola grisea *have been used in combination with *Trichoderma* cellulases to improve the saccharification of cellulose. The study also demonstrated that the recombinant BGL4 from *Humicola grisea* showed strong activity towards cellobiose and the incorporation of the recombinant BGL4 led to improvement in cellulose saccharification by 1.4–2.2 times. Overexpression of recombinant BGL4 gene from *Humicola grisea *in *T. reesei *or *T. viride *has been reported* to *improve the saccharification of cellulose by cellulases complex [43].

 A *β*-glucosidase cloned from *T. reesei* and its expression studies have been reported in *Pichia pastoris* GS115 strain [39]. *T. reesei *produced *β*-glucosidase in very low amounts [27] which acted as a limiting factor in cellulose degradation. To overcome this, it has been reported that a *β*-glucosidase from *T. reesei *(bglI) was over expressed in *Pichia pastoris* GS115 under the control of methanol-inducible alcohol oxidase (AOX) promoter and *S. cerevisiae* secretory signal peptide (a-factor). The expression of *β*-glucosidase in the culture medium has been reported to reach the productivity of 0.3 mg/mL and the maximum activity was reported as 60 U/mL. Furthermore, the protein purification yielded a recombinant *β*-glucosidase of molecular weight 76 kDa, a 1.8-fold purification with 26% yield, and a specific activity of 197 U/mg was achieved. The optimum activity of the enzyme was at 70°C and pH 5.0. 

 Several studies aimed at the improvement of the fungus *T. reesei *for *β*-glucosidase production since the yield is reported to be quite low and it is also required for conversion of cellobiose to glucose which hampers cellulase production. Dashtban and Qin [37] successfully engineered a *β*-glucosidase gene from the fungus *Periconia *spp. into the genome of *T. reesei* QM9414 strain. As compared to the parent strain (2.2 IU/mg), the *T. reesei* strain showed about 10.5-fold (23.9 IU/mg) higher *β*-glucosidase activity after 24 h of incubation. The recombinant enzyme was thermotolerant and was completely active when incubated at 60°C for two hours. Also, a very high total cellulase activity (about 39.0 FPU/mg) was found in comparison to the parent strain which did not show any total cellulase activity at 24 h of incubation. Furthermore, enzyme hydrolysis assay using untreated NaOH or Organosolv pretreated barley straw showed that the recombinant *T. reesei* strains released more reducing sugars compared to the parental strain. Such studies would benefit the bioconversion techniques, namely, biomass conversion using cellulases. 

### 5.3. Bioinformatics Studies

#### 5.3.1. Site Directed Mutagenesis

 Another approach of mutational studies was performed by Lee and coworkers [35] and it showed that induced mutations in the active site of *β*-glucosidase from *T. reesei* lead to improved enzyme activity and thermostability of the enzyme. The study involved mutations in the outer channel of the active site of the enzyme. The mutants, P172L and P172L/F250A showed enhanced enzyme activity in terms of 5.3- and 6.9-fold increase in *K*_*m*_ and *k*_cat_ values towards 4-nitrophenyl-b-D-glucopyranoside (p-NPG) substrate at 40°C as compared to the wild type. Also, L167W or P172L mutations lead to higher thermostability of the enzyme as demonstrated by their melting temperature, Tm. Furthermore, the mutant, L167W, showed an effective synergistic activity together with cellulases in cellulose degradation. These mutational studies hold prospects in engineering enzymes having industrial applications such as biofuel production. 

#### 5.3.2. Biochemical Studies

Several inhibitors were used to study the enzyme activity of *β*-glucosidase from *T. reesei* QM 9414 strain. Diethylpyrocarbonate (DEP) at a concentration above 10 mM completely inhibited the enzyme activity while the presence of substrate or analog protected the enzyme from inactivation. The enzyme showed a pseudo-first-order reaction kinetics, having a second-order rate constant of 0.02 mM^−1^ min^−1^. The presence of 1 M hydroxylamine restored the enzyme activity which indicated the modification of histidine residues. Also, statistical analysis of residual fractional activity compared to the number of modified histidine residues exhibited that presence of one histidine residue is important for catalysis. Other inhibitors of *β*-glucosidase include *p*-hydroxymercuribenzoate which completely inhibited the enzyme at concentration above 2 mM. The modified enzyme when treated with 5,5^'^-dithio-bis (2-nitrobenzoic acid) (DTNB) showed that presence of one cysteine residue was essential for enzyme activity. Also, various other inhibitors like 2-ethoxy-l-ethoxycarbonyl-1,2-dihydroquinoline (EEDQ) were used to study the effect of chemical modifications on enzyme kinetics [79]. 

## 6. Biotechnological Applications of ***β***-Glucosidases

Studies on *β*-glucosidases have been carried out from different sources, namely, microbes, plants, and animals [7–14]. Amongst these, fungal sources are immensely explored due to their better prospects in commercial applications. *β*-glucosidases are promising candidates of glycosyl hydrolase family and catalyze the selective cleavage of glucosidic bonds. The enzyme is found in all living organisms and involved in diverse biological processes, namely, cellular signaling, oncogenesis, host pathogen interactions, degradation of structural and storage polysaccharides, and processes of industrial relevance [26]. Due to the rising significance of *β*-glucosidases in industrial biotechnology, emerging trends focus on the maximum exploitation of this category of enzymes. In plants, the enzyme catalyzes the beta-glucan synthesis during cell wall development, fruit ripening, pigment metabolism, and defence mechanisms [20, 21] while in microorganisms, these are involved in cellulose induction and hydrolysis [80, 81]. In humans and mammals, the enzyme catalyzes the hydrolysis of glucosyl ceramides [82]. Biosynthesis of glycoconjugates such as aminoglycosides, alkyl glucosides, and fragments of phytoalexin-elicitor oligosaccharides which play a role in microbial and plant defence mechanism is an important application of *β*-glucosidases [26]. However, the saccharification of cellulosic biomass for biofuel production is the most extensive area of research and application. The fungal *β*-glucosidases, being an efficient biocatalysts, finds applications in various industrial processes. The major applications of *β*-glucosidases from *Trichoderma species* are as follows.

*Bioethanol Production*. The rising energy demands and depletion of fossil fuels initiated research on alternative sources for energy production. Lignocellulosic biomass is the abundant component of plants and renewable in nature therefore utilized for bioethanol production. Cellulase enzyme complex catalyzes cellulose degradation and comprises of three different enzymes: exoglucanase, endoglucanase, and *β*-glucosidase (BGL) which acts synergistically for complete hydrolysis of cellulose [83, 84]. The initial steps include the cleavage of cellulose fibers by endoglucanase releasing small cellulose fragments which are acted upon by exoglucanase resulting in small oligosaccharides, cellobiose which is hydrolysed into glucose by *β*-glucosidases. The cellulolytic enzyme complex secreted by fungus, *T. reesei* is most widely used in industrial bioethanol applications. The conversion of cellobiose to glucose is regarded as the rate limiting step in bioethanol production from lignocellulosic biomass due to low efficiency and high costs of cellulases. Also, hyper-producing strains of *T.reesei* produce *β*-glucosidase in very low amounts [27]. Alternative methods such as cocultivation fungal strains producing cellulose and *β*-glucosidase, namely, *T. reesei* and *A. phoenics* or *A. niger* was used to enhance the activity of *β*-glucosidase [85]. 

 Several alternatives strategies have been utilized such as heterologous expression of *β*-glucosidase in other systems for enhanced production [85], supplementation of exogenous *β*-glucosidase to the cellulase complex of *T. reesei* [86], engineering *β* glucosidase for overexpression and production [37], promoter use for enhanced expression [74], and site directed mutagenesis [35]. Enzyme preparations consisting of extracellular *β*-glucosidase produced by *T. atroviride *mutants and cellulase producing *T. reesei* were found to be better than commercial preparations for saccharification and of pretreated spruce [87]. Furthermore, studies also indicated that enzyme mixtures from different fungal strains exhibited better activity than commercial preparations namely celluclast 1.5 L, novozyme 188, and accellerase 1000 [86]. Delignified bioprocessings from *Artemisia annua* (known as marc of Artemisia) and citronella (*Cymbopogon winterianus*) have been utilized for bioconversion by six species of *Trichoderma* and cellulase production [88]. Among six species, *T. citrinoviride* was found to be most efficient producer of cellulases and a high amount of *β* glucosidase. Also, *T. virens* was not capable of producing complete cellulase enzyme complex on any test waste or pure cellulose, except on marc of *Artemisia*, where it produced all three enzymes of the complex [89].  Table 2 exhibits various enhancement studies and the possible outcome in terms of fold enhancement obtained for production of *β*-glucosidases from different strains of *Trichoderma*.

## 7. Conclusion

Biofuel production from lignocellulosic biomass comprising cellulose complex is the most important application and accounts for maximum exploitation of enzyme in industrial processes. However, slow enzymatic degradation rate and feed-back inhibition of the enzyme (particularly *β*-glucosidase) limit their commercialization. Current *β*-glucosidase applications involve manipulation strategies such as development of glucose-tolerant *β*-glucosidase and external administration together with other cellulases. Development of mutants and genetic engineering studies is an emerging area with good prospects in enzyme development with desired properties. 

 Commercially, companies such as Novozymes and Genencor have developed cellulolytic enzymes cocktails for biomass hydrolysis such as Cellic series of enzymes [90] and Accellerase series of enzymes [91]. Although, the details of enzyme mixture is not disclosed, but it was assumed that the enzymes preparations were from genetically modified *T. reesei* with high *β*-glucosidase activity. With the tremendous progress on *β*-glucosidases with an aim to improve its production and catalytic activity, it is likely that in near future, these would cease to be a limiting factor in biofuel production. Further, expectedly with the ongoing research efforts in this field, the management of energy crisis and fuel demands to a certain extent would be balanced by the biofuels generation and management.

## Figures and Tables

**Table 1:** Studies on *β*-glucosidase from different strains of *Trichoderma* fungus.

S. no.	*Trichoderma *strain	*β*-glucosidase	Isolation strategies	References
1	*T. citrinoviride *	Extracellular **β**-Glucosidase	Protein purification, biochemical and proteomic characterization	[28]
2	*T. reesei *	TrBgl2	Mutational studies involving active site residues of the enzyme	[35]
3	*T. reesei *QM9414	bgl1	Overexpression of bgl1 from* Periconia* sp. in *T. reesei* QM9414 under *T. reesei* tef1*α* promoter	[37]
4	*Recombinant T. reesei strain, *X3AB1	bgl1	Construction of *T. reesei* strain expressing *A*. *aculeatus* bg1 under control of xyn3 promoter	[38]
5	*T. reesei *	bgl I	Molecular cloning and expression in *Pichia pastoris *	[39]
6	*T. reesei *CL847	BGL1	Protein purification and kinetic characterization	[3]
7	*T. reesei *	*β*-Glucosidase (cel3a)	Molecular cloning and expression in *T. reesei *	[40]
8	*T. ressei *	*β*-Glucosidase BGLII (Cel1A)	Molecular cloning, expression in *E. coli*, and characterization	[41]
9	*T. harzianum *C-4	—	Protein purification and biochemical characterization	[42]
10	*T. reesei *	BGL2	Molecular cloning and expression in *Aspergillus oryzae *	[43]
11	*T. harzianum strain *P1	1,3-**β**-Glucosidase	Protein purification and characterization	[44]
12	*T. reesei* QM9414	Aryl-**β**-D-glucosidase	Protein purification and characterization	[45]
13	*T. viride *	**β**-Gluc I	Protein purification and biochemical characterization	[46]
14	*T. viride* QM9414 mutants	—	Biochemical studies (pH control)	[16]

**Table 2:** Studies comprising of the enhancement strategies used for *β*-glucosidase production.

S. no.	Strain used and enzymes	Enhancement strategies	Conclusion	Reference
1	*Aspergillus aculeatusβ*-glucosidase 1	A recombinant *T. reesei* strain, X3AB1 under the control of xyn3 promoter	63- and 25-fold higher *β*-glucosidase activity against cellobiose	[38]
2	*β*-glucosidase from *Periconia *spp.	Heterologous expression in *T. reesei *	Around 10.5-fold (23.9 IU/mg) higher *β*-glucosidase activity A very high total cellulase activity (about 39.0 FPU/mg)	[63]
3	*T. reesei*, Bgl2	Mutational studies and engineering of active site residues	Mutants, P172L, and P172L/F250A showed enhanced *k*_cat_/*K*_*m*_ and *k*_cat_ values by 5.3- and 6.9-fold Also, mutant L167W had the best synergism with *T. reesei* in cellulosic biomass degradation	[32]
4	*T. reesei *(bglI)	Overexpression in *P. pastoris* GS115 under methanol-inducible alcohol oxidase promoter and *S. cerevisiae* secretory signal peptide.	*β*-glucosidase expression was 0.3 mg/mL and the maximum activity was 60 U/mL	[39]
5	*β*-glucosidase 1 (BGL1)	Use of xyn3 and egl3 promoters through homologous recombination	4.0- and 7.5-fold higher *β*-glucosidase activity	[70]
6	*T. citrinoviride* mutants	Mutational studies, use of ethidium bromide and ethyl methyl sulphonate as mutagens	Secretion of endoglucanase, *β*-glucosidase and cellobiase was found to be 2.14-, 2.10-, 4.09-, and 1.73-fold higher	[28]
7	Thermostable *β*-glucosidase (*cel3a*)	*cel3a* from *Talaromyces emersonii *was expressed in *T. reesei *	High specific activity against *p*-nitrophenyl-*β*-D-glucopyranoside (*V*_max⁡_, 512 IU/mg) and was competitively inhibited by glucose (*k*i, 0.254 mM) and displayed transferase activity	[40]
8	BGL4 from *H. grisea *	Overexpression of BGL4 in *T. reesei *or *T. viride *	Improvement in cellulose saccharification by 1.4–2.2 times	[43]
9	*T. reesei *Rut C-30	Temperature and pH profiling studies	0.02% Tween-80 concentration was optimum, pH 5.0 and temperature (31°C) initially (for 18 h) was optimum for maximum production of cellulase and *β*-glucosidase	[89]

## Abbreviations

CBHI: Cellobiohydrolase I
BGL1: *β*-Glucosidase-1
CBHII: Cellobiohydrolase II
CAZY: Carbohydrate active enzyme database
TrBgl2: *T. reesei* *β*-glucosidase 2
GH1: Glycosyl hydrolase family 1
P(O)NPG: *p*-Nitrophenyl b-D glucopyranoside
*K*_*m*_: Michaelis constant
pI: Isoelectric point
Ki: Inhibitor's dissociation constant
KDa: Kilo dalton
SDS-PAGE: Sodium dodecyl sulfate polyacrylamide gel electrophoresis
MALDI-TOF: Matrix-assisted laser desorption/ionization- time of flight
FPLC: Fast protein liquid chromatography
*V*_max⁡_: maximum rate of reaction
